# Berlin evaluates school tobacco prevention - BEST prevention: study design and methodology

**DOI:** 10.1186/1471-2458-14-871

**Published:** 2014-08-23

**Authors:** Falk Müller-Riemenschneider, Lilian Krist, Christin Bürger, Nanette Ströbele-Benschop, Stephanie Roll, Nina Rieckmann, Jacqueline Müller-Nordhorn, Stefan N Willich

**Affiliations:** Institute for Social Medicine, Epidemiology and Health Economics, Charité Universitätsmedizin, Luisenstrasse 57, 10117 Berlin, Germany; Saw Swee Hock School of Public Health, National University of Singapore, 16 Medical Drive, Singapore, 117597 Singapore; Institute of Nutritional Medicine, University of Hohenheim, Fruwirthstraße 12, 70599 Stuttgart, Germany; Berlin School of Public Health, Charité Universitätsmedizin, Seestraße 73, 13347 Berlin, Germany

**Keywords:** Smoking prevention, Adolescents, Parents, Randomized-controlled trial

## Abstract

**Background:**

The hazardous health effects of smoking are established, but there remains a need to evaluate existing smoking prevention strategies and to increase their effectiveness in adolescents. Strategies focusing on parental attitudes and rule setting have been identified as a potentially effective approach. The present manuscript describes objectives, study design and methodology of the BEST Prevention study.

**Methods/design:**

BEST Prevention is a three-armed cluster randomized-controlled trial among 7^th^ grade (11–16 years) students in Berlin, Germany. Schools were enrolled between 2010 and 2011 and allocated using a centralized randomization list into 1) a student smoking prevention intervention (visit to an established interactive circuit), 2) the same intervention plus a parent intervention, and 3) a control group (visit to an established exercise and nutrition interactive circuit). Students were assessed at baseline, 12 and 24 months via self-report, as well as via carbon monoxide and cotinine in saliva at the 24 month follow-up. Statistical analyses uses multi-level regression models with cluster effects (school and class within school) based on the intention to treat population. Here we report descriptive baseline characteristics of recruited schools, and schools classes. Two schools from the control group dropped out after allocation. Hence, 47 secondary schools from all 12 districts of the city, including 161 school classes and 3023 students are participating in the study. Of those, 2801 students completed the baseline assessment.

**Discussion:**

The present manuscript provides details on the study design and methodology of a large school-based smoking prevention trial in a metropolitan area in Germany. Findings from this study will yield important insight into the long-term effectiveness of specific smoking prevention strategies, also in disadvantaged population groups.

**Trial registration:**

NCT01306552 (January 2011).

## Background

The hazardous health effects of smoking and second hand smoke are well known. Although smoking rates in many industrialized countries have declined over past decades, absolute number of smokers is increasing and so is the absolute mortality attributable to smoking [1–3]. Tobacco use among children and adolescents in many industrialized countries has decreased in recent years, including Germany [4–7]. However, compared to other western countries, smoking rates in Germany are still relatively high which is cause for concern, given the numerous detrimental health effects of smoking [8].

The majority of adult smokers initiate this unhealthy behavior during adolescence, and almost every adult who smokes started smoking before the age of 26 [9]. Targeting children and adolescents is therefore the most appropriate approach to prevent smoking initiation in the first place. School-based prevention strategies have traditionally been an important approach to smoking prevention in children and youths. Particular advantages are that schools offer an almost universal reach of children and youths. In addition, educational strategies fit mutually with schools’ role [10]. However, the effectiveness of such strategies has been mixed and systematic reviews reported limited evidence of the long-term effectiveness of school-based smoking prevention strategies [10, 11].

Parents have a strong influence on their children’s smoking behavior in various ways. For instance, children of parents who smoke are more likely to smoke [12]. Moreover, parental anti-smoking attitudes and rules have shown to be associated with children’s smoking behavior, irrespective of their own smoking behavior [13–15]. Given the at best modest long-term effectiveness of behavioral smoking prevention strategies [10]. and the important role of parents in shaping children’s smoking behavior, parental and family-based intervention strategies have been added to student centered school-based smoking prevention strategies. However, only a limited number of methodologically rigorous studies have investigated the effectiveness of family or parental approaches to smoking prevention and while some reported favorable outcomes, others reported less positive findings [16–19]. Recent systematic reviews of the evidence have suggested that studies incorporating parental or family components could indeed beneficially influence smoking behavior in children and adolescents [11, 20]. In addition to a general paucity of relevant studies these reviews emphasized the fact that the additional effectiveness of parental or family interventions on top of a student targeted smoking prevention strategies was rarely investigated [11, 20].

In Germany, a considerable number of smoking prevention efforts and programs targeted at children and adolescents are available. However, the majority of these activities have never been rigorously evaluated for their efficacy and effectiveness. This is highlighted in systematic reviews of smoking prevention strategies that identified few methodologically rigorous studies from Germany [10, 11]. In addition, there seem to be considerable regional disparities in smoking behavior [21]. Especially in the former eastern parts of Germany and metropolitan areas, such as Berlin, smoking rates tend to be substantially higher compared to average smoking rates. At present, the reasons for these disparities are poorly understood. However, to reduce inequalities in risk taking behavior among adolescents and to subsequently reduce inequalities in health, efforts to target these disadvantaged populations will have to be strengthened. Hence, generally there remains a need to continue developing more effective smoking prevention approaches and to evaluate the effectiveness of individual intervention components, particularly in disadvantaged population groups.

The Berlin Evaluates School Tobacco Prevention - BEST Prevention study was designed to address important research needs. It aims to investigate the long-term comparative effectiveness of a school-based intervention versus a school-based plus parental intervention strategy. The BEST Prevention study targets adolescents in Berlin, a diverse population group with a relatively high proportion being from families with migrant background in a large metropolitan area. The present manuscript describes objectives, study design and methodology of the BEST Prevention study.

## Methods/design

### Objectives

The overall objective of this school-based intervention study is to compare the effectiveness of different smoking prevention strategies among 7^th^ grade students. More specifically this study has three primary objectives:

- To investigate the effectiveness of a combined student-parent intervention to reduce the prevalence of regular smoking (defined as smoking at least one cigarette per week) after two years compared to a control group.
- To investigate the effectiveness of a combined student-parent intervention to reduce the prevalence of regular smoking after two years compared to a student alone intervention.
- To investigate the effectiveness of a student intervention to reduce the prevalence of regular smoking after two years compared to a control group.

Important secondary objectives include:

- To investigate the effectiveness of the interventions with regard to other measures of smoking prevalence (e.g. lifetime smoking prevalence, current smoking status, 12 months prevalence) and in relation to 1 year outcomes
- To investigate smoking status on a subsample of students using objective measures of smoking behavior (carbon monoxide [CO], cotinine in saliva) and the relation between objective and self-reported smoking measures
- To assess the acceptability of the program (e.g. percentage of school principals that agree to support the program, percentage of parents who participate in the parental component, percentage of students participating in the project)
- To assess whether possible intervention effects are moderated by other factors, specifically demographic characteristics (age, gender, individual and neighborhood socio-economic status), type of school (Gymnasium, integrated secondary school), smoking status of friends and family members

### Study population

This study includes 7^th^ grade students from secondary schools throughout Berlin. All 214 secondary schools in the city state of Berlin were approached in 2010. Permission from the senate of education and research had been obtained. Subsequently, school principals and contact teachers in charge of health promotion and smoking prevention at schools from all districts of the city were informed, where possible through workshops about the project and its goals. Secondary schools were subsequently invited to participate in the BEST-Prevention study and were asked to indicate the number of classes participating. All schools that wished to participate provided a letter of interest signed by the school principle. This letter also indicated which and how many 7^th^ grade classes were going to participate in the study. Schools and students were enrolled in the study if the following selection criteria were met.

#### School inclusion criteria

- Participating schools must have 7th grade classes and should not offer an extensive smoking prevention program for their students that includes parental involvement
- Participating schools must agree not to use the student smoking prevention intervention for the duration of the study in case of being randomized to the control group
- Participating schools must agree to have a parents’ night where trained health coaches introduce and discuss the topic of smoking prevention in youth, in case of being randomized to the combined student-parent intervention

##### Student inclusion criteria

- Female or male in 7^th^ grade
- Attends one of the participating schools
- Intellectual and physical ability to make an informed decision about study participation

Approval from the Charité-Universitätsmedizin Berlin institutional review board was obtained and separate signed written informed assent was required from participating students as well as consent from at least one parent/caregiver. Participant information and consent forms were distributed during school classes prior to a second appointment at schools to perform baseline data collection.

### Study design

This study is a three-armed parallel cluster randomized controlled trial among secondary schools with 7^th^ grade classes. As the unit of randomization schools were randomly assigned using a 1:1:1 ratio and a blocked randomization with variable block length, stratified by school type (Gymnasium vs. integrated secondary school) to one of three intervention groups. The randomization sequence was generated using central computer generated randomization lists and allocation was concealed from participating schools.

- Intervention 1:student smoking prevention circuit
- Intervention 2:student smoking prevention circuit plus parent intervention
- Control group:student nutrition and physical activity circuit (without smoking prevention)

Given the nature of the intervention, schools and study participants are not blinded to the assigned intervention. However, data analysis of follow-up outcomes will be blinded with regard to the intervention group.

**Figure 1:**
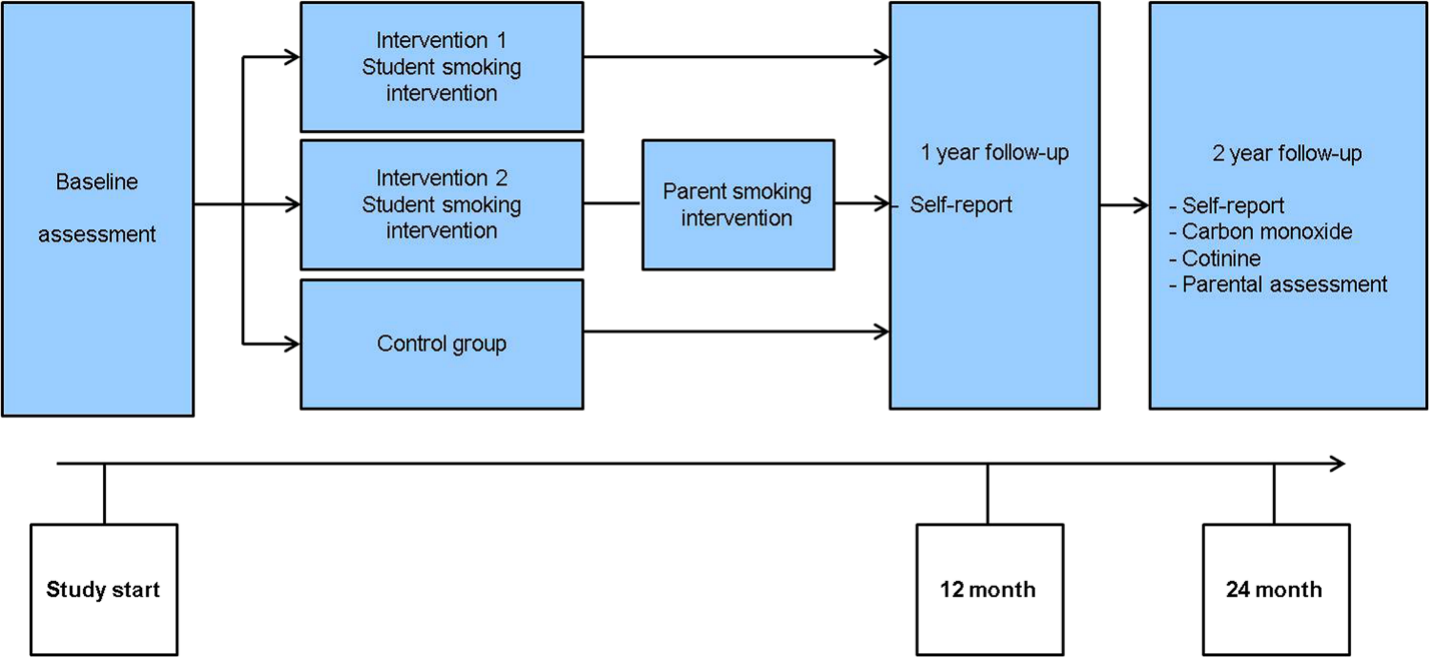
**Study design: three-armed cluster randomized-controlled trial.**

Data collection for each student is conducted at three different time points, including baseline (at the beginning of the 7^th^ grade school year), follow-up 1 (12 month) and follow-up 2 (24 month) (Figure 1). Prior to baseline data collection one additional visit was performed in all school classes in order to inform students about the study and to distribute participant information and consent forms.

### Interventions

#### Intervention 1 - student smoking prevention circuit

Within the first school year school-classes of schools randomized to intervention 1 visited a hands-on smoking prevention circuit with their school class. The circuit (“Rauchst Du noch oder lebst Du schon?” [“Still smoking or already living?”]) was developed and is offered by KARUNA e.V. a non-governmental, non-profit organization for children and youths in need, with the support of the Berlin senate for health and social affairs The aim of the circuit is to inform students about the harmful consequences of tobacco use, to strengthen self-responsibility and self-confidence and to enter into a dialogue with the students. The design of the circuit, the practical approach, and the youth-friendly presentation aim to facilitate the development of a positive non-smoking image among students. In addition, the circuit aims to convey smoking-related preventive knowledge using a game-based approach, including competitions and activities in an age-appropriate and engaging way. Trained moderators lead through six interactive stations of the circuit and an informative billboard (s. Table 1). Overall, completion takes approximately 2 hours. Students learn about the harmful effects of smoking, toxic ingredients of cigarettes, differences between smokers and non-smokers in terms of their health status (atherosclerosis prevalence), loss of smell, breathing capacity and premature aging. The framework for the six stations includes an introduction by the moderator in the form of a presentation (about 20 min) and a final discussion of “unbelievable” facts from the world of tobacco, e.g. from politics and media. (http://www.karuna-prevents.de/index.php).

**Table 1:** **Description of interactive stations of the student smoking prevention circuit**

Interactive station	Content	Activity
1. Addiction	• Education and awareness in relation to the development of nicotine addiction	16 cards showing each a person and a statement have to be allocated to 4 steps symbolizing the stages of addiction:
	• Stages of the development of nicotine/cigarette addiction: Interest → Trial → Habituation → Addiction	Students get points for each correct allocation
2. Knowledge	• Facts/information on tobacco smoke/cigarettes: statistics, dangers, health effects, addiction etc.	Students answer multiple choice questions on a computer
3. Aroma	• Sensory experience: Recognition of different odors	Students have to recognize 8 odors and allocate them to diverse advertisements (e.g. cigarettes, cars, perfumes)
4. Breath	• Sensory experience: Breathing sounds of smokers and non-smokers (and a lion)	Students listen to breathing sounds over a headphone and have to allocate the sounds to a list of answers
5. Toxin Memory	• Relevant toxins in cigarettes and tobacco smoke	Students have to identify pairs of memory cards by allocating a toxic ingredient of cigarettes to the product where it is normally used, e.g.: Arsenic - rodent control; plumb – battery; naphthalene - insecticides)
6. Arterio-sclerosis	• Development of atherosclerosis and its consequences	Students have to pump water through two water tubes. One is normal, the other one constricted to show differences in the circulatory system of smokers and non-smokers.
	• Blood flow in non-smokers and in long-term smokers with arteriosclerosis	Followed by quiz.
Information billboard	• Physical appearance of smokers	As part of this billboard students try to recognize smokers/non-smokers by physical appearance.

School classes complete the smoking prevention circuit in small groups of 3–5 participants. Each station includes a quiz or a competition to be completed. Based on the results groups receive points and the total number of points identifies the winning group. The prize of the winning group is participation in a virtual aging tool of one student in the group in order to show aging effects of smoking. The photo of the respective student will be taken and manipulated (by April® Face Aging Software) to show the student’s appearance in 20 years in two versions (if he/she had/had not smoked). In addition, school classes are enrolled in a Berlin wide competition. In each grade the class with the most points wins the KARUNA - Champion Award.

#### Intervention II - student plus parent smoking prevention intervention

School-classes of schools randomized to the student-parent intervention group also visited the same smoking prevention circuit. In addition, an intervention for children’s parents is offered. The parent intervention takes place during the first year of the study and consists of two parts. First, during the first routine parent’s night, parents take part in an educational program about smoking prevention in children and youths. The intervention is based upon the program “Eltern stärken” [strengthening parents] and is provided by trained health coaches and consists of a 30 minute presentation. The program follows a normative approach and provides parents with knowledge and skills to address smoking behavior in adolescents and their children. Key topics addressed are:

- Current facts about smoking behavior in adolescents, including smoking initiation
- Evidence regarding parental influence on adolescent smoking
- Facts about smoking cessation in youths
- The evidence regarding parental attitudes towards smoking, parental smoking behavior and parental rules towards smoking behavior
- Discussion with parents

The second part of the parental intervention consists of mailed follow-up including informational materials during the second year of the study (about 8 months after the parent’s night). This follow-up contact re-emphasized information and strategies taught during the event.

#### Control group - student healthy nutrition and exercise circuit

School-classes of schools randomized to the control group participated in the healthy nutrition and exercise circuit offered by KARUNA e.V. (“Kinderleicht gesund zu leben” [“Healthy living – as easy as pie”]). The circuit follows a similar methodological approach to the smoking prevention circuit, but has no smoking related parts. It targets student’s knowledge to make healthy decisions with a focus on diet and exercise. A trained moderator leads the small groups in about 2 hours through the five stations summarized in Table 2 (for more information on the contents of the nutrition and exercise circuit see http://www.karuna-prevents.de/index.php).

**Table 2:** **Description of interactive stations of the student healthy nutrition and exercise circuit**

Interactive station	Content	Activity
1. Healthy shopping	• Development of skills for healthy shopping (balance of carbohydrates, fat and protein)	Students have to choose food out of 90 products which they believe are healthy. They get points for a well-balanced selection of healthy foods.
2. Flavor bar	• Food flavors	Students taste 7 water solved flavors and have to allocate them to food products, eg.: Strawberry – strawberry ice-cream; mint – cough drops)
3. Nutrition pyramid	• Nutritional recommendations	Students get 14 cubes with pictures of food items and have to build the nutrition pyramid.
4. Exercise	• Exercise and energy expenditure	Students cycle on a bicycle ergometer in order to burn as many calories as possible.
		The moderator explains the amount and kind of food that corresponds to burned calories.
5. Knowledge	• Knowledge on nutrition and exercise	Students complete a quiz about healthy nutrition and health promoting exercise.

The completion of the circuit in small groups is similar to the smoking prevention circuit. Also, based on accumulated points a group winner will be identified. As in the smoking prevention circuit, school classes will be enrolled in a Berlin wide competition. In each grade the class with the most points wins the KARUNA - Champion Award.

### Outcomes and data collection

#### Primary outcome

The primary outcome is the proportion of regular smokers (smoking at least one cigarette per week) assessed by self-report at 24 months (final follow-up).

#### Secondary outcomes include

- Other measures of smoking behavior (e.g. lifetime prevalence, 30 day prevalence, 12 months prevalence, number of cigarettes, cannabis use)
- Other health behaviors (e.g. alcohol, nutrition, physical activity)
- Parental attitudes, rules and smoking behavior
- Cotinine and CO measurements on a random subsample of students

During all three assessment points (baseline, 12 month and 24 month) relevant outcomes are assessed using self-administered questionnaires during school classes from all study participants. Two to three trained members of the study team are available during data collection and are responsible for all aspects of the data collection process within school classes. At each school, contact teachers supported the implementation of the data collection and intervention implementation. A query management was implemented by the research team to track data collection and intervention implementation at participating schools. To coordinate the assessments at schools, contact teachers were approached and reminded multiple times and eg. via email, phone, and through the principal in order to reduce the number of drop-outs at the cluster level (school and school class). Students who had provided complete written informed consent but were not available during data collection in schools received mailed questionnaires together with free return envelopes for completion at home.

The study questionnaire for adolescents was developed and pilot tested in a way to allow comparisons with existing and widely comparable questionnaires investigating adolescent health behavior and health. It includes questions related to socio-demographics, smoking and other health behaviors, such as alcohol consumption, nutrition, physical activity and sedentary behaviors, as well as height and weight. In addition to smoking behavior, the questionnaire addresses various other issues related to adolescent smoking, including smoking behavior of family members and peers, parental rules and attitudes towards smoking, as well as peer pressure.

About two months before the final follow-up of their children, all parents received a brief self-administered questionnaire via mail. Primary purpose of this questionnaire was to determine whether parents/caregivers whose children were allocated to Intervention 2 attended the parent’s night. In addition, the questionnaire addresses parental smoking behavior, awareness about their child’s smoking behavior, attitudes and rules towards smoking of their child or at their home, as well as a brief evaluation of the parental intervention. Parents whose children were allocated to Intervention 1 and Control group (parents who did not receive the parent intervention) received the same questionnaire except for items related to the evaluation of the parental intervention.

In addition to self-reported outcomes in students, the main outcome, smoking behavior, is assessed objectively at the final follow-up. Assessments are conducted in the class-room during questionnaire assessments. Measurements are conducted and handled in such a way that findings are not visible to other students or the teacher. Objective outcome assessments include CO measurements from exhaled air and saliva based cotinine measurements. Assessments are conducted on a random subsample of students from 15 schools. Students from 6 schools (2 in each intervention group) undergo CO measurements. The CO content [22] is measured in ppm (part per million) using the Bedfont Smokerlyzer Micro +. Smoking behavior according to this tool is classified as follows: 0–4 non-smoker, 5–6 dangerous exposure, 7–10 smoker, 11+ heavy smoker. Another subsample of students from 6 schools (2 in each intervention group) provides saliva samples via passive drool to measure cotinine values using a NicAlert® dipstick. The NicAlert® test yields a semi-quantitative measure of cotinine based on a colorimetric immunoassay reaction. The test strip displays seven zones that represent a range of cotinine levels from 0 (0-10 ng/ml) to 6 (>1000 ng/ml). Results are recorded as values from 0 to 6; a result ≥ 1 indicated tobacco use [23]. In a further subsample of students from 3 schools (1 in each intervention group) students provide both objective outcome measurements described above.

Data management at the Institute for Social Medicine, Epidemiology and Health Economics is conducted according to standard operating procedures. Questionnaire data is entered by study personnel into a password protected database. Data quality is checked by means of plausibility tests and implausible data are compared against the original questionnaires. Double data entry of a random sample of participants is conducted to control the rate of data entry errors. Access to the data is only permitted to specific members of the research team.

### Sample size determination

The analysis is following a hierarchical testing procedure. In a first step, intervention 2 (student smoking prevention circuit plus parent intervention) will be compared to the control group with regard to the primary outcome (proportion of regular smokers at 24 months). Only if step 1 is significant at the 5% level (two-sided), will step 2 be tested confirmatively (otherwise all following analyses will be considered explorative). The second step involves two hypotheses: Intervention 1 (student smoking prevention circuit) compared to control; and Intervention 2 compared to Intervention 1, each with regard to the primary outcome. The sample size determination is based on the second step (having a smaller assumed intervention effect, thus requiring a higher sample size than step one) with the two hypotheses tested equally (significance level alpha 0.025 each). Taking into account the cluster design with an assumed intra-cluster correlation coefficient for schools (ICC) of 0.001 and a power of 80%, 15 (14.7 and 13.3 precisely for the two hypotheses) schools (=clusters) are required in each of the three arms to detect a 5% difference in the proportion of regular smokers after two years (assumptions: intervention 1: 30%, intervention 2: 25%, and control group: 35%). Since we assume on average 60 participating students per school, this yields a total of 15 × 3 × 60 = 2700 required students. Assuming a drop-out rate of about 20% this yields a targeted sample size of n = 3375 students to be randomized.

### Statistical analysis

In general, multi-level regression models (Generalized Linear Mixed Models (GLMM)) with cluster effects (school and class within school) will be used for all statistical analyses. For the primary analysis, a logit model will be used, which will be adjusted for smoking status at baseline, based on the intention to treat (ITT) population. Testing will be hierarchical as described above with an overall level of significance of 5% (two-sided). All further analyses will be considered explorative.

Secondary analysis of the primary endpoint include models with adjustment for smoking status at baseline and other baseline variables in case of relevant imbalances between treatment groups. In addition, missing values will be imputed as sensitivity analyses. A per-protocol (PP) population will be defined and analyzed in a similar manner.

Secondary endpoints will be analyzed within similar frameworks as applicable.

All analyses will be specified in detail in a statistical analysis plan (SAP) which will be finalized prior to data analysis. Data analysis is conducted with the SAS for Windows, Version 9.3 or higher (SAS Institute, Cary, NC, USA) or other software.

## Results

### Recruitment process and distribution of participating schools and students

**Figure 2:**
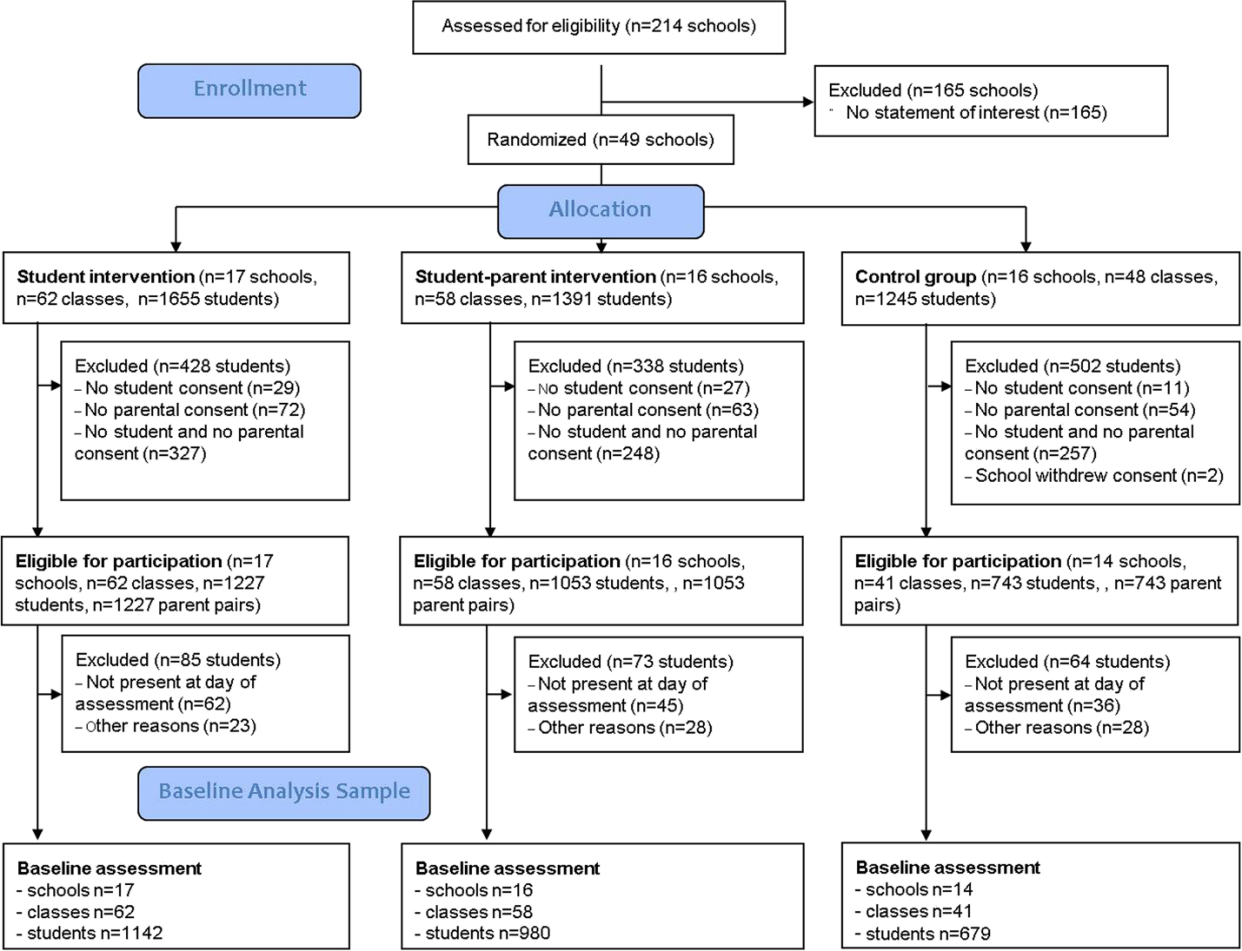
**Study-flow up to baseline assessment.**

**Figure 3:**
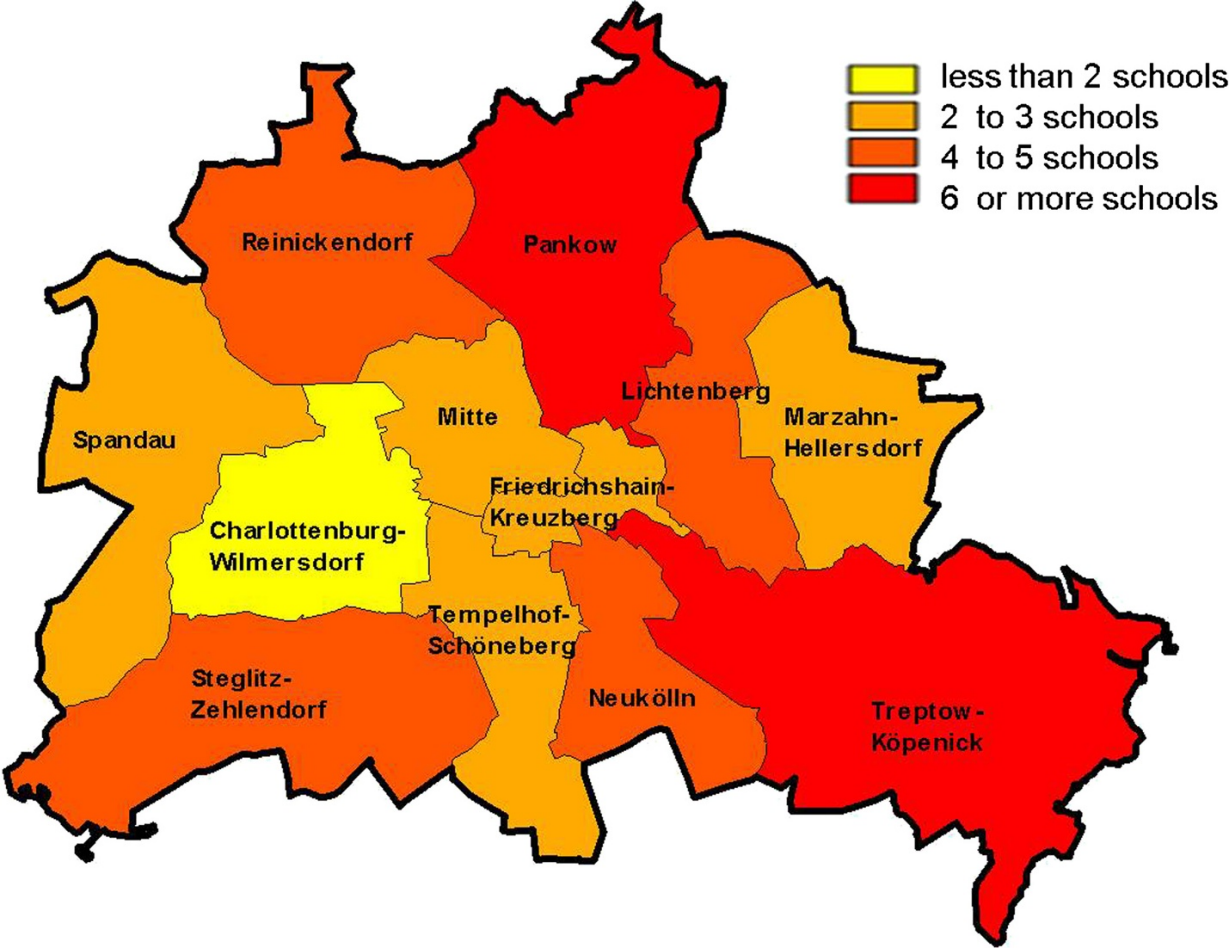
**Distribution of participating schools across all 12 districts of Berlin.**

Schools, school classes and study participants were recruited over a period of two school years. Out of all 214 secondary schools in Berlin that were approached in 2010, a total of 49 secondary schools were enrolled over two recruitment waves and randomized. Two schools withdrew despite their initial commitment after being randomized to control group, leaving a total of 47 participating schools. During the school year 2010/11 (wave 1) 32 schools were enrolled. Further 15 schools were enrolled during the school year 2011/12 (wave 2)). For details of the school and participant recruitment process see Figure 2. Schools were recruited from all 12 districts of the city state of Berlin (s. Figure 3). With regard to the school type, 32 are considered integrated secondary schools while 15 schools are high schools (“Gymnasien”).

Within these 47 schools, the total number of recruited school classes is 161, reflecting an average number of school classes per school of 3.4 (SD: 1.8). The total number of students meeting all selection criteria and providing written informant assent and parental consent is n = 3023, reflecting the total study population. For a detailed description of the allocation of schools, school classes and students see Table 3. Out of those, 2801 participants completed the baseline study questionnaire (s. Figure 2).

**Table 3:** **Distribution of schools, school classes and students, as well as cluster sizes overall and by intervention groups**

	Overall	Student intervention	Student-parent intervention	Control group
**Total schools**	47	17 (36%)	16 (34%)	14 (30%)
** Integrated secondary school**	32	11 (34%)	11 (34%)	10 (31%)
** Gymnasium**	15	6 (40%)	5 (33%)	4 (27%)
**School classes**	161	62 (42%)	58 (31%)	41 (27%)
**Students**	2801*	1142 (41%)	980 (35%)	679 (24%)
**Students per school type**				
** Gymnasium**	1173* (41.9%)	525 (46.0%)	394 (40.2%)	254 (37.4%)
** Integrated secondary school**	1628* (58.1%)	617 (54.0%)	586 (59.8%)	425 (62.6%)
**Classes per school (mean ± SD)**	3.4 ± 1.8	3.7 ± 1.8	3.6 ± 1.8	3.0 ± 1.9
**Students per school (mean ± SC)**	59.6 ± 35.3*	67.2 ± 36.2	61.3 ± 38.2	48.5 ± 30.1
**Students per class (mean ± SD)**	17.4 ± 6.3*	18.4 ± 5.5	17.2 ± 6.5	16.2 ± 7.1

*SD*: standard deviation, *Based on the number of students completing the baseline questionnaire.

## Discussion

The BEST-Prevention study is among the largest randomized controlled trials in Germany that investigates the effectiveness of smoking prevention strategies among adolescents [10]. It was designed to address important and unresolved issues in the context of behavioral smoking prevention strategies. Findings will be of importance for a number of reasons: Firstly, the BEST-Prevention study targets specific intervention components and addresses the additional effectiveness of a feasible parental smoking prevention strategy to reduce smoking rates among adolescents, which few studies have done so far [11, 20]. Secondly, it targets adolescents at increased risk of smoking due to their diverse background and their residence in a large metropolitan area in Germany. Thirdly, this study assesses the long-term effectiveness of a smoking prevention intervention.

Despite recruitment challenges due to the implementation of a school reform in Berlin in 2010, which reduced the number of potentially eligible schools by about 50%, the targeted number of 45 schools and 161 school classes was achieved or even exceeded. However, with 3023 students meeting all selection criteria, the estimated sample size was not fully met.

Although our study did not aim to enroll a representative population of 7^th^ graders in Berlin we were able to recruit schools and students from all 12 districts of the city. This ensures that schools and students are located in districts and neighborhoods with diverse characteristics and SES. While we recruited schools, school classes and students from all these districts, the distribution of those does not fully reflect the general distribution across the city of Berlin. For instance, certain districts are overrepresented in terms of the relative number of schools and students enrolled, while others underrepresented. In terms of the type of school, there are 121 integrated secondary schools and 93 “Gymnasien” in Berlin. Within the BEST-Prevention study we enrolled 32 integrated secondary schools and 15 Gymnasien, reflecting a somewhat different distribution. Our population based sample in a major metropolitan area in Germany and its diversity will offer the opportunity to investigate the impact of various individual family characteristics, as well as neighborhood SES on smoking and other health behaviors. This will make findings highly relevant to populations which are frequently not adequately reached through prevention efforts.

Outcomes of the present school-based RCT are assessed at baseline, 12 month and 24 month through self-report, similar to many previous school-based smoking prevention studies [10, 11, 20]. However, self-report has its limitations and can result in over- or underreporting of relevant behaviors. Another strength of the present study is that in addition to self-report, main study outcomes will be assessed using objective measures, namely CO and cotinine measurements. This will provide valuable additional information to strengthen conclusions based on self-report outcomes.

## Conclusion

In summary, the present manuscript provides an overview of the study design and methodology of a large school-based smoking prevention cluster randomized controlled trial in a metropolitan area in Germany. Recruitment of the BEST-prevention study was successful in enrolling targeted numbers of schools and school classes, although the number of students was somewhat lower than planned. Moreover, enrolled schools and students represent all districts of the city. Findings from our study will soon provide valuable information with regards to the acceptability and effectiveness of specific smoking prevention strategies also in disadvantaged population groups. Furthermore, the additional effectiveness of strategies targeting parents will be determined.

### Ethics

Approval from the Charité-Universitätsmedizin Berlin institutional review board was obtained.
